# Supplementary material for: Chromothripsis during telomere crisis is independent of NHEJ, and consistent with a replicative origin
Source: Genome Res. 2019 May;29(5):737–49. doi: 10.1101/gr.240705.118 (PMC6499312; doi:10.1101/gr.240705.118)
Supplement: Supplemental Material [file supp_gr.240705.118_Supplemental_file_1.zip › contigs/annotated_contigs/DB116/contig.2.DB116_length_517_mean_cov_5.79883945841.docx]

**DB116_length_517_mean_cov_5.79883945841**

```
ACATAATTTGATTTCAAATTATATAAAAGCATGCTTTATATATTAAAATGTCAGTAGATTCATCTATTTAATCAAGTTTCTTATTGGTT
 >chr11:80570441-80570648 - E=6e-110
GACTATTGTACCAAACACTTAGCTAGTCACTGAGGATTCAATGGGGAATGAAGTGGACATGGTCCCTACGTTTGGAAAATTTATTATTT

AATGGTGAAACAAACATTAAAAAACAG|CA|TGTACAAATGTTTCCATTCAAAACTTACATTCTAGTGTGAGTTTCCCTGGCAGATCAA
                            >chr11:80571551-80571863 + E=3e-176
GTGCTCATCTTATATATATATATTTTTTCCATTTTCTCTTTCAGATTACTTCCCAGATTTTCAACACCATCTATCCTCCTCTCTGTCTG

CCAAGCCTTTTATTTGATATTTAGCATATGACATTAACTCCATGGCTAATTGACTTCATAAGACATAACCAAAATGAGGTGTATTTTTC

TAATGAAAGACCACATTTGAAAATGCAAAATAAGATAACCTATAATAACAATGACTCATATCTGGGGAAGCAGC
```
